# Impact of S1P Mimetics on Mesenteric Ischemia/Reperfusion Injury

**DOI:** 10.3390/ph13100298

**Published:** 2020-10-09

**Authors:** Francesco Potì, Carmine Giorgio, Irene Zini, Jerzy-Roch Nofer, Valentina Vivo, Simone Palese, Vigilio Ballabeni, Elisabetta Barocelli, Simona Bertoni

**Affiliations:** 1 Department of Medicine and Surgery, University of Parma, via Gramsci 14, 43126 Parma, Italy; francesco.poti@unipr.it; 2 Food and Drug Department, University of Parma, Parco Area delle Scienze 27/a, 43124 Parma, Italy; carmine.giorgio@unipr.it (C.G.); irene.zini@gmail.com (I.Z.); valentina.vivo@unipr.it (V.V.); simone.palese@studenti.unipr.it (S.P.); vigilio.ballabeni@unipr.it (V.B.); elisabetta.barocelli@unipr.it (E.B.); 3 Institute of Clinical Chemistry and Laboratory Medicine, University Medical Center Hamburg-Eppendorf, 20251 Hamburg, Germany; j.nofer@uke.de

**Keywords:** sphingosine-1-phosphate, gut hypoxia-reperfusion, FTY720, ozanimod, inflammation, S1P_1_

## Abstract

Mesenteric ischemia/reperfusion (I/R), following the transient deprivation of blood flow to the gut, triggers an acute flogistic process involving the disruption of endothelial and epithelial barriers integrity, the activation of immune cells, and the abundant release of inflammatory mediators. Among them, the lipid mediator sphingosine-1-phosphate (S1P) is involved in maintaining epithelial and endothelial barrier integrity and in governing the migration of immune cells through the interaction with S1P_1–5_ receptors. Therefore, the present work aims to investigate the involvement of S1P signaling in intestinal I/R-induced injury by studying the effects of FTY720, the non-selective S1P_1,3–5_ agonist, and comparing them with the responses to ozanimod, selective S1P_1,5_ agonist, in a murine model of gut I/R. Intestinal edema, gut and lung neutrophil infiltration, and oxidative stress were evaluated through biochemical and morphological assays. The collected results highlight the protective action of FTY720 against the inflammatory cascade elicited by mesenteric I/R injury, mainly through the control of vascular barrier integrity. While these beneficial effects were mimicked by ozanimod and can be therefore attributed largely to the effects exerted by FTY720 on S1P_1_, the recruitment of myeloid cells to the injured areas, limited by FTY720 but not by ozanimod, rather suggests the involvement of other receptor subtypes.

## 1. Introduction

Sphingosine-1-phosphate (S1P) is a biologically active lysosphingolipid generated by the sphingosine kinase-catalyzed phosphorylation of sphingosine. S1P mediates and activates cell survival, proliferation, migration, and differentiation, and accomplishes critical tasks in maintaining epithelial and endothelial barrier integrity and governing the migration of immune cells. The pleiotropic physiological effects of S1P are mediated by binding to five ubiquitously expressed cell-surface G-protein-coupled receptors (S1P_1–5_). As a consequence, the pharmacological targeting of the SKs/S1P/S1P-receptor axis has been proposed for the management of several patho-physiological processes, including cancer as well as cardiometabolic, chronic inflammatory, and auto-immune disorders [1]. Recently, the role of the SKs/S1P/S1P-receptor pathway in the development of gastrointestinal pathologies and cancer has been extensively reviewed by Sukocheva et al. [2], who especially focused on its participation to the development and progression of gastrointestinal pathologies, like inflammatory bowel diseases (IBDs) and cancers of the digestive tract, characterized by an underlying flogistic processes.

Fingolimod (FTY720), a potent and non-selective S1P_1,3–5_ agonist, was the first orally active modulator approved for the treatment of relapsing remitting multiple sclerosis. Upon phosphorylation by sphingosine kinase 2 (SK2), FTY720 induces the internalization and degradation of S1P_1_ in T-cells and the inhibition of their trans-endothelial migration. This leads to the blockade of immune cell egress from lymphoid organs towards high S1P levels in the circulation and the inflammatory sites and to potent immunosuppressive action [3]. Accordingly, apart from multiple sclerosis, FTY720 is under clinical investigation for a number of indications sharing aberrant immune responses, such as inflammatory bowel diseases (IBDs), psoriasis, rheumatoid arthritis, amyotrophic lateral sclerosis, diabetes, and the systemic inflammatory response syndrome (SIRS) [4]. Of note, SIRS can be a dramatically fatal consequence of ischemia–reperfusion (I/R) that may occur in different regions and wherever it happens, triggers an acute flogistic process involving the disruption of endothelial barrier integrity and the activation of immune cells along with the abundant release of inflammatory mediators [5]. In the I/R-induced experimental injury, FTY720-mediated protection was seen in renal [6,7], hepatic [8], and lung [9] districts and lung damage after trauma/hemorrhagic shock was attenuated [10]. However, FTY720 has never been tested in mesenteric I/R injury so far. Therefore, the aim of the present study was to investigate the effects of FTY720 against the local and systemic inflammatory responses induced by intestinal I/R in mice. Yet, FTY720 lacks selectivity towards the S1P_1_ receptor, exactly the subtype involved in lymphocyte trafficking and the endothelial barrier permeability, which are key elements in the development of I/R insult. Accordingly, we asked whether the effects of FTY720 could be mimicked by ozanimod, S1P_1_ and S1P_5_ agonist just clinically approved for multiple sclerosis [11] and currently under clinical investigation for IBDs [3]. Since S1P_5_ is mainly expressed in the brain [4,12], we assumed that the effects of ozanimod in mesenteric I/R should be mainly attributed to S1P_1_ activation.

## 2. Results

### 2.1. S1P Mimetics Decreased Vascular Permeability during Intestine I/R

**Figure 1:**
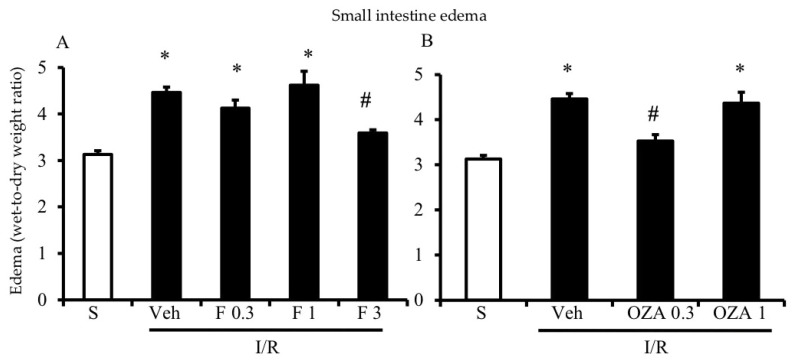
Effects of S1P modulators on I/R-induced plasma extravasation. Edema, expressed as the wet-to-dry weight ratio, of intestinal tissues excised from S mice (white columns) and I/R mice administered with vehicle (Veh), FTY720 (F) 0.3–3 mg/kg i.v. (**A**) or ozanimod (OZA) 0.3, 1 mg/kg s.c. (**B**) (*n* = 6–10 animals per group). * *p* < 0.05 vs. S mice; ^#^ *p* < 0.05 vs. Veh mice, one-way analysis of variance followed by Bonferroni’s post-test.

As evidenced by a significantly higher gut edema in I/R mice as compared to controls, intestinal vascular permeability was markedly increased by the interruption of mesenteric blood flow for 45 min, followed by 5 h reperfusion (Figure 1). Compared with vehicle-treated I/R mice, both FTY720 and ozanimod significantly reduced vascular leakage. FTY720 was effective at the highest tested dose (Figure 1A), whereas ozanimod decreased gut edema only when administered at the lower dose (Figure 1B).

### 2.2. FTY720 but Not Ozanimod Mitigated I/R-Induced Recruitment of Leukocytes

**Figure 2:**
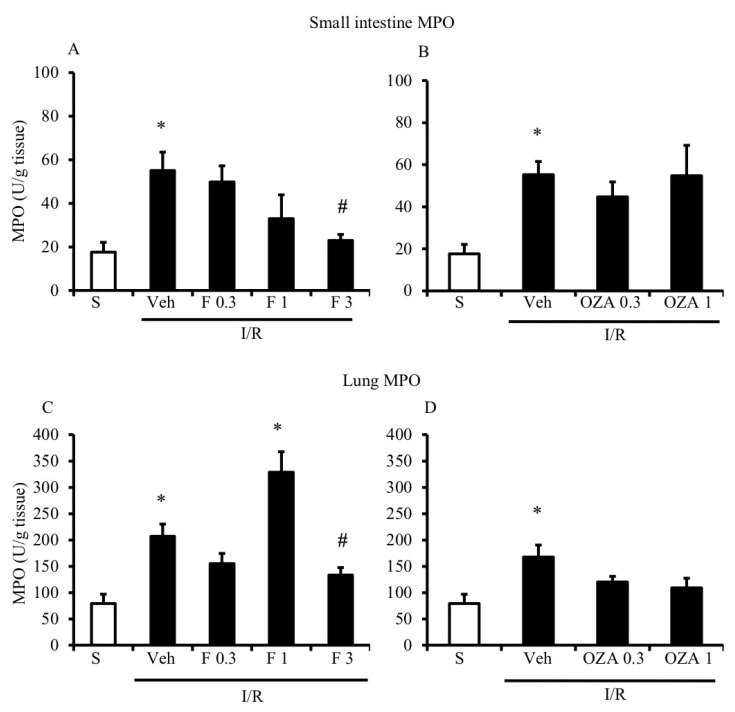
Effects of S1P modulators on ischemia/reperfusion (I/R)-induced neutrophil infiltration. Myeloperoxidase (MPO) activity in the small intestine (**A**,**B**) and lung (**C**,**D**) tissues excised from S mice (white bar) and from I/R mice administered with vehicle (Veh), FTY720 (F) 0.3–3 mg/kg i.v. (**A**,**C**) or ozanimod (OZA) 0.3, 1 mg/kg s.c. (**B**,**D**) (*n* = 6–10 animals per group). * *p* < 0.05 vs. S mice; ^#^ *p* < 0.05 vs. Veh mice, one-way analysis of variance followed by Bonferroni’s post-test.

A strong increase in myeloperoxidase (MPO) activity, which is characteristic for I/R injury and considered predictive of neutrophil recruitment, was determined both in the small intestine and lung tissues after the occlusion of the superior mesenteric artery (SMA) for 45 min followed by 5 h reperfusion (Figure 2).

**Figure 3:**
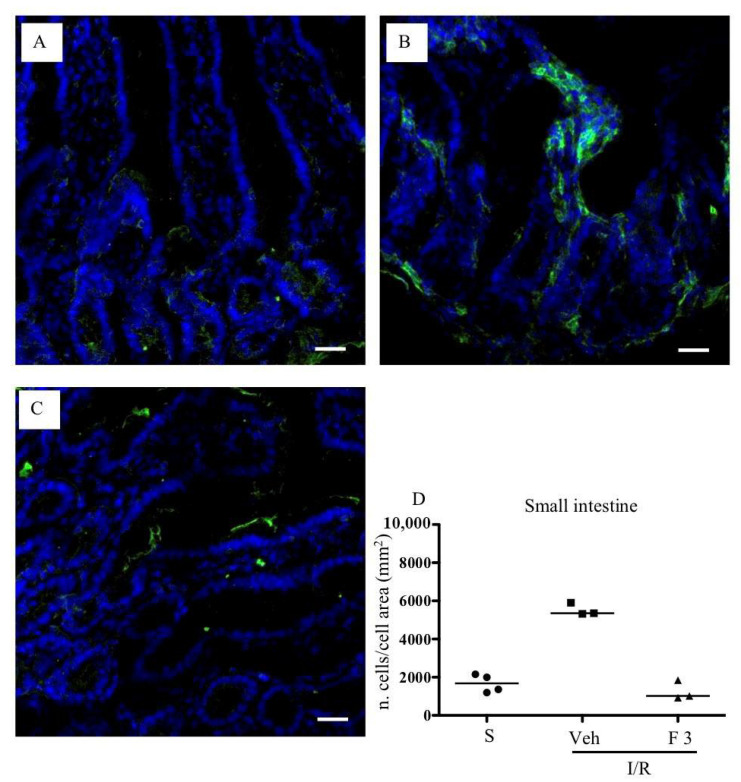
Effects of FTY720 on I/R-induced CD11b^+^ cell recruitment in the small intestine. Representative immunofluorescence-stained sections visualizing CD11b^+^ cells (green fluorescence) in intestinal tissues excised from S mice (**A**) and from I/R mice administered with vehicle (**B**) or FTY720 3 mg/kg i.v. (**C**). Images were taken at 63× magnification through the oil immersion objective (scale bar: 25 μm). Sections were counterstained with DAPI for nuclear morphology (blue fluorescence). (**D**) Ratio of the number of CD11b^+^ cells divided by the cell total area in sections of intestinal tissues excised from vehicle-treated S mice (*n* = 4) and I/R mice administered with vehicle (Veh) (*n* = 3) or with FTY720 3 mg/kg i.v. (F 3) (*n* = 3) (horizontal bar at the median value).

**Figure 4:**
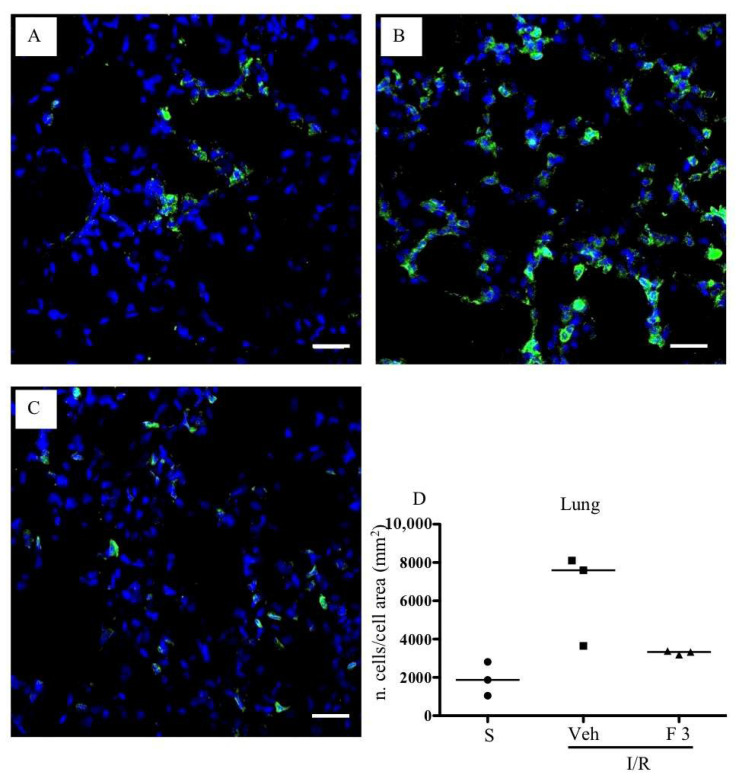
Effects of FTY720 on I/R-induced CD11b^+^ cells recruitment in the lung. Representative immunofluorescence-stained sections visualizing CD11b^+^ cells (green fluorescence) in lung tissues excised from S mice (**A**) and from I/R mice administered with vehicle (**B**) or FTY720 3 mg/kg i.v. (**C**). Images were taken at 63× magnification through the oil immersion objective (scale bar: 25 μm). Sections were counterstained with DAPI for nuclear morphology (blue fluorescence). (**D**) Ratio of the number of CD11b^+^ cells divided by the cell total area in sections of lung tissues excised from vehicle-treated S mice (*n* = 3) and I/R mice administered with the vehicle (Veh) (*n* = 3) or with FTY720 3 mg/kg i.v. (F 3) (*n* = 3) (horizontal bar at the median value).

As shown in Figure 2A,B, FTY720 was able to significantly reduce MPO activity at the highest tested dose in the small intestine, while ozanimod was inactive in this respect. Likewise, in the lungs, ozanimod produced only a slight and not significant tendency towards dampening MPO activity (Figure 2D), while FTY720 was able to consistently reduce that, when administered at the highest dose (Figure 2C). At the same dose, FTY720 was also assessed for its ability to prevent the I/R-induced recruitment of myeloid cells into gut and lung tissues, evidenced through their direct visualization with a confocal microscope. Data are presented in Figure 3 and Figure 4: they indicate that, albeit not significantly, the treatment with the highest dose of FTY720 attenuated the accumulation of myeloid cells triggered by SMA occlusion both in the small intestine (Figure 3) and in the lungs (Figure 4).

### 2.3. S1P Mimetics Failed to Affect Lipoperoxidation during Intestine I/R

In our experimental model of mesenteric I/R, malondialdehyde (MDA) levels, which reflect the extent of oxidative stress, were increased, though not significantly, in intestinal tissues excised from I/R mice as compared to the controls. None of the treatments used in this study were able to attenuate the process of lipoperoxidation triggered by the transient occlusion of SMA (Table 1).

### 2.4. Mesenteric I/R Did Not Affect Mesenteric Lymph Nodes (MLNs) Lymphocytes Count

Given the documented ability of FTY720 in preventing the lymphocyte egress from peripheral lymph nodes, but not from the spleen [13], and the close proximity of MLNs to the small intestine, we asked whether the number of lymphocytes in mesenteric lymph nodes (MLNs) could be affected by mesenteric I/R and by the treatment with FTY720: this would help to highlight the potential participation of lymphocyte migration to the I/R-induced injury and to the beneficial effects of FTY720.

**Figure 5:**
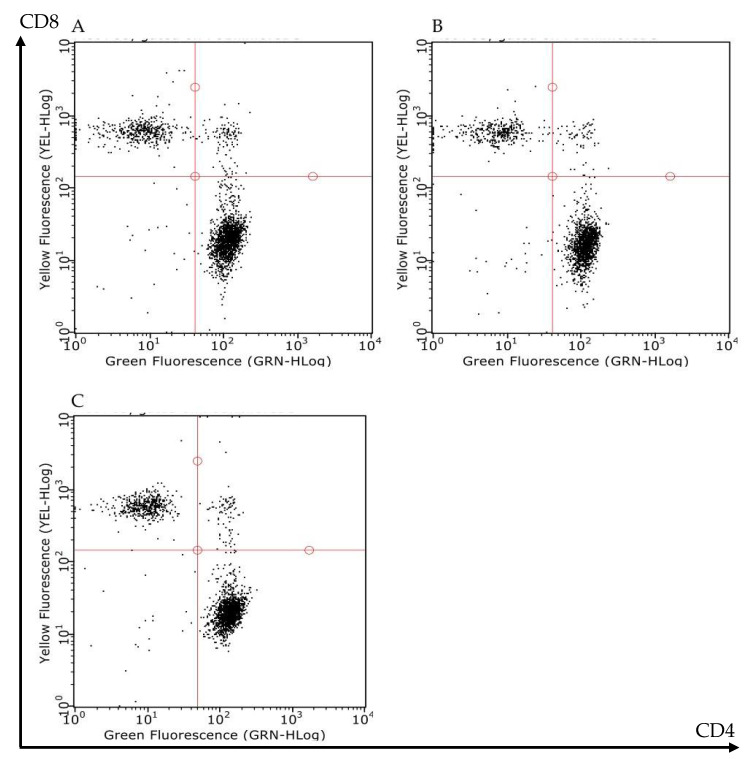
Effects of FTY720 on the T lymphocytes count in MLNs. Representative flow cytometry dot plots showing CD4^+^CD8- (lower-right corner) and CD4-CD8^+^ (upper-left corner) T lymphocytes of MLNs excised from S mice (**A**) and I/R mice administered with vehicle (**B**) or with FTY720 3 mg/kg i.v. (**C**).

MLN T lymphocytes subpopulations were counted by flow cytometry in S and I/R mice administered with vehicle or with FTY720 3 mg/kg. Mesenteric I/R did not produce any change in the number of MLN total cells or in the number of CD4^+^ and CD8^+^ T lymphocytes. Likewise, the administration of the S1P modulator did not overtly modify the MLN lymphocytes count in I/R mice (Table 2 and Figure 5).

## 3. Discussion

The transient interruption of blood flow to a tissue triggers a cascade of self-fueling events involving the activation of endothelial and innate immune cells and the synthesis/release of a multitude of both pro- and anti-inflammatory mediators leading to morphological and functional alterations. Several studies identified S1P among the cell-derived substances locally released during the I/R injury [14]. However, the specific contribution of S1P to the I/R injury development is controversially discussed. Both the native and chaperone-bound S1P as well as the pharmacological S1P receptor mimetics, including FTY720 and selective S1P_1_ agonists, were repeatedly demonstrated to attenuate the I/R injury in heart [15], brain [16], lung [9,17], liver [8], kidneys [7,18], testes and ovaries [19]. On the other side, S1P was implicated in the propagation of the I/R-induced organ damage under particular experimental conditions. For instance, S1P_3_ activation was found to potentiate the I/R injury in the liver, while S1P_3_ deletion was protective in kidneys [20]. In addition, the elimination of SK1, which is the major source for local S1P release, reduced the extent of I/R injury in the liver and other organs [21].

The discrepant results regarding the role of S1P in the pathophysiology of I/R injury prompted us to investigate the potential protective effects of the non-selective S1P_1,3–5_ agonist/functional antagonist FTY720 in mesenteric I/R-induced injury, a model involving the transient interruption of blood flow to the small intestine, in which FTY720 has never been tested thus far. Moreover, to shed additional light on the potential mechanism accounting for the protective action of FTY720, its effects were compared to those of the S1P_1,5_ selective agonist ozanimod. 

Our study shows for the first time that both FTY720 and ozanimod reduced the I/R-induced increase in vascular permeability in the mesenteric area in the murine model of I/R-mediated injury. Given the marginal expression of S1P_5_ outside of the central nervous system [4,12], it is conceivable to attribute the protective action of ozanimod in the mesenteric I/R to the activation of S1P_1_ receptors. Similarly to the present study, the beneficial effects exerted by this compound in chronic inflammatory diseases, such as multiple sclerosis and inflammatory bowel disease, were largely explained by its interaction with S1P_1_ and not with S1P_5_: S1P_1_ is therefore considered its primary therapeutic target. With respect to FTY720, the involvement of other S1P receptor subtypes in mediating its favorable effects on intestine permeability cannot be dismissed. However, previous studies regarding the modulatory effects exhibited by FTY720 on histological and functional injuries induced by I/R in kidneys [6,7,10], liver [8], and lungs [9] have consistently pointed to the activation of S1P_1_ receptors as an underlying mechanism pivotal to attenuate the deleterious consequences of vascular occlusion, likely through maintaining the vascular barrier integrity and the reduction of vascular leakage. 

In our experimental conditions, the I/R-induced increase in vascular permeability in the mesenteric area was mitigated by FTY720 at the highest dose used (3 mg/kg) and in a similar manner, by ozanimod when administered at the lowest dose (0.3 mg/kg): indeed, ozanimod displayed beneficial effects only in a very narrow range dose, showing an inverse dose–response relationship. Several reasons may account for the different potency order exhibited by the two compounds. Besides the diverse routes of administration, FTY720 being intravenously administered and ozanimod subcutaneously injected, distinct pharmacodynamic properties could contribute to the differences in the observed effective dose. If we consider simply the in vitro affinity towards S1P_1_ receptors, ozanimod and FTY720-P, the biologically active-phosphorylated derivative of FTY720, displays comparable agonist activity at sub-nanomolar concentrations [22]. However, other aspects could come into play to make things more intricate: first of all, only a fraction of the injected dose of FTY720 is converted to its pharmacologically active principle FTY720-P by SK2 [23]. It is possible that, during the period of mesenteric I/R that follows its intravenous administration, FTY720 phosphorylation is delayed, slowed down or blunted. Therefore, we cannot exclude that FTY720 administration would lead to concentrations of its active metabolite comparable to those produced by ozanimod, in the time frame of I/R, only when performed at much higher doses. Moreover, while moving from lower to higher doses, ozanimod might first activate S1P_1_, thereby promoting the endothelial barrier integrity [24], and then desensitize the S1P_1_-mediated intracellular signaling following receptor internalization [3], and in so doing, losing its beneficial actions.

With regard to the leukocyte recruitment, FTY720 was able to limit the accumulation of neutrophils and monocytes/macrophages within both the intestinal and pulmonary areas, as evidenced both by biochemical and immunofluorescence analysis, while ozanimod showed substantial inactivity in this respect. Suggestively, receptor subtypes other than S1P_1_ and S1P_5_ are involved in controlling the migration of myeloid cells promoted by mesenteric I/R. Of note, the critical roles of S1P_3_ and S1P_4_ receptors in controlling the adhesion to endothelial cells, the trafficking and activation of immune cells under inflammatory conditions have been postulated by several investigations [25,26]. Recently, in an in vivo model of lipopolysaccharide (LPS)-induced pulmonary inflammation, S1P and FTY720 lowered the leukocyte infiltration into the lungs in a way that was mimicked by a selective S1P_4_ agonist. Interestingly, the decrease in neutrophil migration could be explained by the blockade of leukotrienes biosynthesis caused by the 5-lipoxygenase inhibition brought about by the S1P_4_ receptor activation [27]. With regard to the S1P_3_ receptor activation, FTY720 has also been demonstrated to downregulate the in vitro expression of adhesion molecules on endothelial cells through S1P_3_ via PI3K activation [28], a feature possibly reducing immune cell transmigration into inflamed areas.

As regards the contribution of T cells to I/R-induced injury, following SMA occlusion, T lymphocytes have been shown to adhere to endothelial cells in higher number and to promote neutrophil recruitment in the small bowel [29]: blocking their egress thanks to FTY720 towards the inflammatory sites could therefore play a beneficial action. The findings we collected did not suggest any possible influence of MLN lymphocytes on the alterations caused by I/R nor any effect of FTY720 on their migration from lymph nodes. Although the trafficking of lymphocytes from MLN has been left apparently unmodified by the I/R and by S1P modulator, we cannot exclude that lymphocytes exiting from other lymphoid tissues may participate to the increase in vascular permeability and myeloid accumulation triggered by gut I/R, leaving open the question concerning their involvement in the modulation of I/R-injury by S1P mimetics.

In conclusion, the present results seem to confirm the protective action of FTY720 against the inflammatory cascade elicited by mesenteric I/R injury, mainly through the control of vascular barrier integrity. While these beneficial effects were mimicked by ozanimod and can therefore be largely attributed to the effects exerted by FTY720 on S1P_1_, the recruitment of the myeloid cells to the injured areas, which was limited by FTY720 but not by ozanimod, could suggest rather the involvement of other S1P receptors, such as S1P_3_ and S1P_4_.

## 4. Materials and Methods

### 4.1. Experimental Procedures

All the experiments were authorized by the local Animal Care Committee “Organismo preposto al benessere degli animali” and by the Italian Ministry of Health (Protocol Number 307/2017-PR) and were performed according to the Guiding Principles in the Care and Use of Animals (DL26/2014). All appropriate measures were taken to avoid or soothe the pain or discomfort of animals. Experiments were performed on male adult Swiss mice (22–27 g; Charles River Laboratories, Calco, Italy) housed under standard conditions (12:12 h light–dark cycle, 22–24 °C, food and water available ad libitum) and fasted 12 h before the experiment with free access to water. All animals were euthanized by CO_2_ inhalation.

### 4.2. Drugs

FTY720 was purchased from Sigma-Aldrich (St. Louis, MO, USA) and ozanimod was bought from Cayman Chemical (Ann Arbor, Michigan, USA). Myeloperoxidase was purchased from Calbiochem. All other chemicals of reagent grade were obtained from Sigma Aldrich if not otherwise indicated. The day of the experiment, stock solution of FTY720 was diluted with saline solution and ozanimod was dissolved in carboxymethylcellulose 0.5% *w*/*v* to the desired concentration.

### 4.3. Ischemia/Reperfusion

Mice were anaesthetized with pentobarbital (70 mg⋅kg^−1^ i.p.) and a midline laparotomy was performed. The superior mesenteric artery (SMA) was identified and isolated, and a small vascular clamp was applied [30]. The bowel was covered with a surgical gauze moistened with warm 0.9% saline in order preserve the tissue. After 45 min, the clamp was removed and the reperfusion was allowed. Following 5 h reperfusion, the animals were euthanized by CO_2_ inhalation. Animals subjected to I/R were randomly assigned to the following experimental groups: vehicle (Veh) (10 mL/kg i.v.) (*n* = 30); FTY720 0.3 mg/kg (*n* = 6) 1 mg/kg (*n* = 7), 3 mg/kg (*n* = 8) i.v. 5 min prior to ischemia; ozanimod (OZA) 0.3 mg/kg (*n* = 7), 1 mg/kg (*n* = 6) s.c. 15 min prior to ischemia. Preliminary studies showed that, when tested at 3 mg/kg, ozanimod significantly worsened the clinical conditions of mice and the experiments were therefore interrupted. The doses of the compounds were chosen on the basis of the literature [9,22,31]. Sham-operated mice (S) underwent the same surgical manipulations, except for SMA occlusion, and received saline (0.9% NaCl *w*/*v*), i.v., or carboxymethylcellulose (0.5% *w*/*v*), s.c., respectively, 5 min or 15 min prior to laparotomy (*n* = 8).

This study was performed using experimental blocks composed of 8 or 10 mice that were randomly assigned to four or five groups of treatment (vehicle-treated I/R mice—Veh—were present in each experimental block), each one encompassing two animals.

Due to possible seasonal variability, Veh mice were repeated periodically all over the study in order to verify the attainment of a constant degree of I/R injury, thus explaining the bigger size of the Veh experimental group with respect to the other groups. 

### 4.4. Myeloperoxidase Activity

From each mouse, the entire small intestine was longitudinally cut and each half was subjected either to MPO activity assay or malondialdehyde level determination (see method below), while excised lungs were entirely employed to determine the MPO activity. MPO activity, the index of tissue neutrophil infiltration, was assessed according to the o-dianisidine assay [32]. Intestinal and pulmonary specimens were homogenized (1:10 *v*/*v*) in a solution containing aprotinin 1 μg/mL dissolved in 100 mM potassium phosphate buffer (pH 7.4) and centrifuged for 20 min at 7000× *g* at 4 °C. Pellets were re-homogenized in five volumes of 50 mM potassium phosphate buffer (pH 6.0) containing 0.5% hexadecylthrimethyl–ammonium bromide and aprotinin 1 μg/mL. The samples were subjected to three cycles of freezing and thawing, and then centrifuged for 30 min at 5000× *g* at 4 °C. An aliquot (0.1 mL) of the supernatant was then allowed to react with 0.9 mL of a buffer solution containing o-dianisidine (0.167 mg/mL) and 0.0005% H_2_O_2_. The rate of change in absorbance was measured spectrophotometrically at 470 nm (Jenway, mod. 6300, Dunmow, Essex, England). One unit of MPO was defined as the quantity of enzyme degrading 1 μmol of peroxide per minute at 25 °C. Data were expressed in units per gram of dry weight tissue.

### 4.5. Malondialdehyde Assay

The production of the oxidative stress derivative malondialdehyde (MDA) was used as marker of lipoperoxidation. Gut MDA levels were estimated according to the method devised by Ohkawa [33]. The small intestine was homogenized in 1.15% KCl solution (1:10 *v*/*v*). An aliquot (0.1 mL) of the homogenate was added to a solution containing 0.2 mL of 8.1% sodium dodecyl sulphate, 1.5 mL of 20% acetic acid (pH 3.5), 1.5 mL of 0.8% thiobarbituric acid and 0.7 mL distilled water. Samples were then heated for 1 h at 95 °C and centrifuged at 3000× *g* for 10 min. The absorbance of the supernatant was spectrophotometrically determined at 532 nm (Jenway, mod. 6300, Dunmow, Essex, England). Data were expressed in nmol per gram of dry weight tissue.

### 4.6. Vascular Permeability

The increased capillary permeability of the intestinal mucosa, expressed as increased tissue water content, was determined using the wet-to-dry weight ratio according to Moore-Olufemi et al. [34]. Intestinal samples about 1 cm in length, located at the jejunal level, were excised from each mouse, cut along the anti-mesenteric border and weighted (wet weight) before placing the tissues in an oven set to 60 °C. Tissues were dried over 72 h and dry weights were measured. Tissue water content was determined and expressed as the ratio (wet weight–dry weight)/dry weight. 

### 4.7. Flow Cytometric Assays

#### 4.7.1. Isolation of Mesenteric Lymph Nodes (MLNs)

After suppression, the harvesting of the whole MLN chain located in the mesentery of the proximal colon was performed in S mice or in mice subjected to I/R, either administered with FTY720 3 mg/kg, i.v., or with vehicle (10 mL/kg i.v.). MLNs were rinsed with PBS, cleaned of vascular and adipose tissues, mechanically dispersed through a 100 μm cell-strainer in Hank’s balanced salt solution (HBSS) containing 5% FCS. The obtained suspension was centrifuged at 1500 RCF for 10 min at 4 °C, the pellet was washed with HBSS containing 5% FCS and re-suspended in 3 mL cell staining buffer. The cellular suspension was stained with fluorescent antibodies.

#### 4.7.2. Immunofluorescent Staining

Two hundred microliters (200 µL) of cellular suspension from MLNs was incubated with IgG1-Fc (1µg/10^6^ cells) for 10 min in the dark at 4 °C to block non-specific binding sites for antibodies. The following antibodies were used for the subsequent fluorescent staining: phycoerythrin–cyanine 5 (PE–Cy5) conjugated anti-mouse CD3ε (0.25 µg/10^6^ cells, catalog number 15-0031, lot number B226301), Fluorescein isothiocyanate (FITC) anti-mouse CD4 (0.25 µg/10^6^ cells, catalog number 100406, lot number B210488), PE anti-mouse CD8a (0.25 µg/10^6^ cells, catalog number 100708, lot number B190687). Cells were incubated with antibodies for 1 h in the dark at 4 °C, washed with PBS to remove excessive antibody and suspended in cell staining buffer (FCS 2%, NaN_3_ 0.05%, EDTA 2 mM in PBS) to perform flow cytometry analysis. Staining through propidium iodide (PI), a membrane non-permeable DNA intercalating agent, excluded by viable cells and emitting red fluorescence once bound to DNA, allowed to evaluate the viability of the cellular suspension. Cells were incubated with PI 10 µg/mL for 1 min in the dark, at room temperature, and immediately processed to flow cytometry analysis. Only PI^-ve^ cells were included in the analysis. 

Samples were analyzed using InCyte™ software (Merck Millipore, Darmstadt, Germany) and cell populations were defined as follows: lymphocytes after gating in the forward scatter (FSC)-side scatter (SSC) plot (FSC low: SSC low); T lymphocytes (CD3^+^ lymphocytes); CD4^+^ helper T lymphocytes (CD3^+^CD4^+^CD8^−^ lymphocytes); CD8^+^cytotoxic T lymphocytes (CD3^+^CD8^+^CD4^−^ lymphocytes). Percentages were reported to the total number of MLN cells of each mouse and the number of cells per population was calculated in each experimental group.

### 4.8. Histology and Immunofluorescence Analysis

In order to perform a preliminary immunohistological study on myeloid cells recruitment, the samples of small intestine and lungs were harvested from additional vehicle-treated S and I/R mice and from I/R mice treated with FTY720 3 mg/kg and were subjected to immunofluorescence analysis. After euthanasia, the caudal vena cava was cut and mice were exsanguinated; without opening the thorax, the trachea was cannulated with PE-50 tubing and the lungs were fixed in situ with 10% neutral buffered formalin. Intestinal tissues were excised, flushed with saline and immersion-fixed in 10% neutral buffered formalin. After fixation for 45 min at room temperature, samples of the lungs and small intestine were incubated overnight at 4 °C in a 20% sucrose-phosphate buffered solution. Successively, the organs were cut in small pieces, embedded in tissue-tek optimal cutting temperature (OCT) compound and sectioned in a cryostat (Reichert-Jung Frigocut 2800E). Eight micrometers-thick cryosections were air dried, blocked, and incubated overnight at 4 °C with a primary antibody against murine CD11b (AbD Serotec) antigen (1:100), marker for both monocytes/macrophages and neutrophils. Then, the sections were washed, incubated with DyLight 488 secondary antibody (Novus Biologicals) for 1 h at room temperature. After washing, the sections were counterstained and mounted with a 4′,6-diamidino-2-phenylindole (DAPI)-containing medium (Fluoroshield, Sigma-Aldrich). Images were captured by confocal microscopy (TCS SP2, Leica) and analyzed with Fiji Software [35]. Briefly, for each acquired field, the positive cells were manually counted (on average 3–4 fields/sample). The surface area covered by the cell nuclei was quantified through a computer-assisted threshold function (Fiji) and used as a proxy of cellularity for data normalization. Hence, the results were expressed as ratio of the number of positive cells divided by the cell total area. The average value of the ratio determined for the small intestine and the lungs in each animal was pooled with those determined for the other animals of the same experimental group and the median value was determined.

## Figures and Tables

**Table 1:** Effects of S1P modulators on I/R-induced lipoperoxidation. Malondialdehyde (MDA) levels in small intestinal tissues excised from S mice and from I/R mice administered with vehicle, FTY720 (0.3–3.0 mg/kg i.v.) or ozanimod (0.3, 1.0 mg/kg s.c.) (*n* = 6–10 animals per group). Data are expressed as the mean value ± s.e.m.

Treatment	MDA (nmol/g)
S	304.1 ± 60.6
I/R + vehicle	686.9 ± 88.4
I/R + FTY720 0.3 mg/kg	1813.0 ± 315.6 *^,#^
I/R + FTY720 1.0 mg/kg	525.3 ± 76.7
I/R + FTY720 3.0 mg/kg	1020.0 ± 225.8
I/R + ozanimod 0.3 mg/kg	539.1 ± 79.9
I/R + ozanimod 1.0 mg/kg	871.9 ± 253.6

* *p* < 0.05 vs. S mice; ^#^ *p* < 0.05 vs. vehicle-treated I/R mice, one-way analysis of variance followed by Bonferroni’s post-test.

**Table 2:** Mesenteric lymph nodes (MLNs) lymphocytes count. Number of total lymphocytes, CD4^+^CD8- and CD4-CD8^+^ T lymphocytes in MLNs excised from S mice and from I/R mice administered with vehicle or FTY720 3.0 mg/kg i.v. (*n* = 5 independent values per group). Data are expressed as the mean value ± s.e.m.

Treatment	Total Lymphocytes (×10^5^)	CD4^+^CD8- (×10^5^)	CD4-CD8^+^ (×10^5^)
S	25.5 ± 0.8	13.4 ± 2.0	4.9 ± 1.0
I/R + vehicle	24.0 ± 3.9	13.6 ± 2.0	4.1 ± 0.7
I/R + FTY720 3.0 mg/kg	24.3 ± 3.0	13.8 ± 1.7	3.9 ± 0.5

## References

[1] Maceyka M., Harikumar K.B., Milstien S., Spiegel S. (2012). Sphingosine-1-phosphate signaling and its role in disease. Trends Cell Biol..

[2] Sukocheva O.A., Furuya H., Ng M.L., Friedemann M., Menschikowski M., Tarasov V.V., Chubarev V.N., Klochkov S.G., Neganova M.E., Mangoni A.A. (2020). Sphingosine kinase and sphingosine-1-phosphate receptor signaling pathway in inflammatory gastrointestinal disease and cancers: A novel therapeutic target. Pharmacol. Ther..

[3] Stepanovska B., Huwiler A. (2020). Targeting the S1P receptor signaling pathways as a promising approach for treatment of autoimmune and inflammatory diseases. Pharmacol. Res..

[4] Huwiler A., Zangemeister-Wittke U. (2018). The sphingosine 1-phosphate receptor modulator fingolimod as a therapeutic agent: Recent findings and new perspectives. Pharmacol. Ther..

[5] Bertoni S., Ballabeni V., Barocelli E., Tognolini M. (2018). Mesenteric ischemia-reperfusion: An overview of preclinical drug strategies. Drug Discov. Today.

[6] Troncoso P., Ortíz M., Martínez L., Kahan B. (2001). FTY 720 prevents ischemic reperfusion damage in rat kidneys. Transplant. Proc..

[7] Awad A.S., Ye H., Huang L., Li L., Foss F.W., Macdonald T.L., Lynch K.R., Okusa M.D. (2006). Selective sphingosine 1-phosphate 1 receptor activation reduces ischemia-reperfusion injury in mouse kidney. Am. J. Physiol. Physiol..

[8] Man K., Ng K.T., Lee T.K.W., Lo C., Sun C.K., Li X.L., Zhao Y., Ho J.W., Fan S.T. (2005). FTY720 Attenuates Hepatic Ischemia-Reperfusion Injury in Normal and Cirrhotic Livers. Arab. Archaeol. Epigr..

[9] Stone M.L., Sharma A.K., Zhao Y., Charles E.J., Huerter M.E., Johnston W.F., Kron I.L., Lynch K.R., Laubach V.E. (2015). Sphingosine-1-phosphate receptor 1 agonism attenuates lung ischemia-reperfusion injury. Am. J. Physiol. Cell. Mol. Physiol..

[10] Bonitz J.A., Son J.Y., Chandler B., Tomaio J.N., Qin Y., Prescott L.M., Feketeova E., Deitch E.A. (2014). A Sphingosine-1 Phosphate Agonist (FTY720) Limits Trauma/Hemorrhagic Shock–Induced Multiple Organ Dysfunction Syndrome. Shock.

[11] Lamb Y.N. (2020). Ozanimod: First Approval. Drugs.

[12] O’Sullivan S., Dev K.K. (2017). Sphingosine-1-phosphate receptor therapies: Advances in clinical trials for CNS-related diseases. Neuropharmacology.

[13] Chiba K., Matsuyuki H., Maeda Y., Sugahara K. (2006). Role of sphingosine 1-phosphate receptor type 1 in lymphocyte egress from secondary lymphoid tissues and thymus. Cell. Mol. Immunol..

[14] Raza Z., Saleem U., Naureen Z. (2020). Sphingosine 1-phosphate signaling in ischemia and reperfusion injury. Prostaglandins Other Lipid Mediat..

[15] Karliner J.S. (2008). Sphingosine kinase regulation and cardioprotection. Cardiovasc. Res..

[16] Sanchez T. (2016). Sphingosine-1-Phosphate Signaling in Endothelial Disorders. Curr. Atheroscler. Rep..

[17] Ding R., Han J., Tian Y., Guo R., Ma X. (2011). Sphingosine-1-Phosphate Attenuates Lung Injury Induced by Intestinal Ischemia/Reperfusion in Mice: Role of Inducible Nitric-Oxide Synthase. Inflammation.

[18] Park S.W., Kim M., Kim M., D’Agati V.D., Lee H.T. (2011). Sphingosine kinase 1 protects against renal ischemia–reperfusion injury in mice by sphingosine-1-phosphate1 receptor activation. Kidney Int..

[19] Henry L., Fransolet M., Labied S., Blacher S., Masereel M.-C., Foidart J.-M., Noël A., Nisolle M., Munaut C. (2016). Supplementation of transport and freezing media with anti-apoptotic drugs improves ovarian cortex survival. J. Ovarian Res..

[20] Bajwa A., Huang L., Kurmaeva E., Gigliotti J.C., Ye H., Miller J., Rosin D.L., Lobo P.I., Okusa M.D. (2015). Sphingosine 1-Phosphate Receptor 3–Deficient Dendritic Cells Modulate Splenic Responses to Ischemia-Reperfusion Injury. J. Am. Soc. Nephrol..

[21] Qiang G.-H., Wang Z.-X., Ji A.-L., Wu J.-Y., Cao Y., Zhang G., Zhang Y.-Y., Jiang C. (2019). Sphingosine kinase 1 knockout alleviates hepatic ischemia/reperfusion injury by attenuating inflammation and oxidative stress in mice. Hepatobiliary Pancreat. Dis. Int..

[22] Scott F.L., Clemons B., Brooks J., Brahmachary E., Powell R., Dedman H., Desale H.G., Timony G., Martinborough E., Rosen H. (2016). Ozanimod (RPC1063) is a potent sphingosine-1-phosphate receptor-1 (S1P1) and receptor-5 (S1P5) agonist with autoimmune disease-modifying activity. Br. J. Pharmacol..

[23] Billich A., Bornancin F., Dévay P., Mechtcheriakova D., Urtz N., Baumruker T. (2003). Phosphorylation of the Immunomodulatory Drug FTY720 by Sphingosine Kinases. J. Biol. Chem..

[24] Radeva M.Y., Waschke J. (2017). Mind the gap: Mechanisms regulating the endothelial barrier. Acta Physiol..

[25] Brinkmann V. (2007). Sphingosine 1-phosphate receptors in health and disease: Mechanistic insights from gene deletion studies and reverse pharmacology. Pharmacol. Ther..

[26] Olesch C., Ringel C., Brüne B., Weigert A. (2017). Beyond Immune Cell Migration: The Emerging Role of the Sphingosine-1-phosphate Receptor S1PR4 as a Modulator of Innate Immune Cell Activation. Mediat. Inflamm..

[27] Fettel J., Kühn B., Guillen N.A., Sürün D., Peters M., Bauer R., Angioni C., Geisslinger G., Schnütgen F., Zu Heringdorf D.M. (2018). Sphingosine-1-phosphate (S1P) induces potent anti-inflammatory effects in vitro and in vivo by S1P receptor 4-mediated suppression of 5-lipoxygenase activity. FASEB J..

[28] Imeri F., Blanchard O., Jenni A., Schwalm S., Wünsche C., Živković A., Stark H., Pfeilschifter J., Huwiler A. (2015). FTY720 and two novel butterfly derivatives exert a general anti-inflammatory potential by reducing immune cell adhesion to endothelial cells through activation of S1P3 and phosphoinositide 3-kinase. Naunyn-Schmiedeberg’s Arch. Pharmacol..

[29] Shigematsu T., Wolf R., Granger D.N. (2002). T-lymphocytes modulate the microvascular and inflammatory responses to intestinal ischemia-reperfusion. Microcirculation.

[30] Ebertoni S., Arcaro V., Vivo V., Rapalli A., Tognolini M., Cantoni A.M., Saccani F., Flammini L., Domenichini G., Ballabeni V. (2014). Suppression of inflammatory events associated to intestinal ischemia–reperfusion by 5-HT1A blockade in mice. Pharmacol. Res..

[31] Daniel C., Sartory N., Zahn N., Geisslinger G., Radeke H.H., Stein J. (2007). FTY720 ameliorates Th1-mediated colitis in mice by directly affecting the functional activity of CD4+CD25+ regulatory T cells. J. Immunol..

[32] Krawisz J.E., Sharon P., Stenson W.F. (1984). Quantitative assay for acute intestinal inflammation based on myeloperoxidase activity. Assessment of inflammation in rat and hamster models. Gastroenterology.

[33] Ohkawa H., Ohishi N., Yagi K. (1979). Assay for lipid peroxides in animal tissues by thiobarbituric acid reaction. Anal. Biochem..

[34] Moore-Olufemi S.D., Kozar R.A., Moore F.A., Sato N., Hassoun H.T., Cox C.S., Kone B.C. (2005). Ischemic preconditioning protects against gut dysfunction and mucosal injury after ischemia/reperfusion injury. Shock.

[35] Schindelin J., Arganda-Carreras I., Frise E., Kaynig V., Longair M., Pietzsch T., Preibisch S., Rueden C., Saalfeld S., Schmid B. (2012). Fiji: An open-source platform for biological-image analysis. Nat. Methods.

